# Solubility of α-synuclein species in the L62 mouse model of synucleinopathy

**DOI:** 10.1038/s41598-024-56735-6

**Published:** 2024-03-14

**Authors:** Karima Schwab, Mandy Magbagbeolu, Franz Theuring, Charles R. Harrington, Claude M. Wischik, Gernot Riedel

**Affiliations:** 1 School of Medicine, Medical Sciences and Nutrition, University of Aberdeen, Forester Hill, Aberdeen, AB25 2ZD UK; 2 Institute of Pharmacology, Charité-Universitätsmedizin Berlin, Hessische Str. 3-4, 10115 Berlin, Germany; 3 TauRx Therapeutics Ltd., 395 King Street, Aberdeen, AB24 5RP UK

**Keywords:** Alpha-synuclein, Parkinson’s disease, Mouse model, Protein aggregation, Protein solubility, Neuroscience, Neurology

## Abstract

The accumulation of α-synuclein (α-Syn) into Lewy bodies is a hallmark of synucleinopathies, a group of neurological disorders that include Parkinson’s disease (PD) and dementia with Lewy bodies (DLB). Small oligomers as well as larger fibrils of α-Syn have been suggested to induce cell toxicity leading to a degenerative loss of neurones. A richer understanding of α-Syn aggregation in disease, however, requires the identification of the different α-Syn species and the characterisation of their biochemical properties. We here aimed at a more in-depth characterisation of the α-Syn transgenic mice, Line 62 (L62), and examined the deposition pattern and solubility of human and murine α-Syn in these mice using immunohistochemical and biochemical methods. Application of multiple antibodies confirmed mAb syn204 as the most discriminatory antibody for human α-Syn in L62. Syn204 revealed an intense and widespread immunohistochemical α-Syn labelling in parietal cortex and hippocampus, and to a lower level in basal forebrain and hindbrain regions. The labelled α-Syn represented somatic inclusions as well as processes and synaptic endings. Biochemical analysis revealed a Triton-resistant human α-Syn pool of large oligomers, a second pool of small oligomers that was not resistant to solubilization with urea/Triton. A third SDS-soluble pool of intermediate sized aggregates containing a mixture of both, human and mouse α-Syn was also present. These data suggest that several pools of α-Syn can exist in neurones, most likely in different cellular compartments. Information about these different pools is important for the development of novel disease modifying therapies aimed at α-Syn.

## Introduction

Alpha-synuclein (α-Syn) is a small protein characterised by a lysine-rich N-terminus and an aggregation-prone non-amyloidal component (NAC) region. The N-terminus is crucial for interactions with membranes and other proteins, while the NAC region is essential for α-Syn aggregation^1,2^. Physiological α-Syn exists as an unfolded monomer having an apparent molecular weight of ~ 14 kDa on gels under denaturating conditions, while it adopts an extended conformation under native conditions that results in an anomalous migration behaviour of ~ 60 kDa (for review see^3^). Physiological α-Syn plays a crucial role in synaptic soluble N-ethylmaleimide-sensitive factor attachment protein receptor (SNARE) complex assembly and neurotransmitter release^4–6^. Deletion of synuclein is associated with the development of progressive neurological impairments, while its loss of function/gain of toxicity due to e.g. aggregation is implicated in the pathogenesis of synucleinopathies, neurodegenerative disorders that include Parkinson’s disease (PD) and dementia with Lewy bodies (DLB)^7,8^. A unique deposition pattern of the intracellular α-Syn inclusions is seen across synucleinopathies, where they occur in subcortical neurones in PD but predominantly in cortical brain areas in DLB^9–12^. Aggregation of α-Syn is toxic and leads to the degenerative loss of neurones^13^. However, whether the aggregation of α-Syn into larger fibrils or into smaller oligomers is more harmful is still a subject of debate^14^. To further our understanding of α-Syn in disease requires the examination of the different α-Syn pools/species. While immunohistochemical methods are useful to demonstrate the distribution pattern of α-Syn inclusions across the brain and within cells, biochemical methods combined with differential centrifugation are indispensable tools to address the solubility and the size of aggregated protein species.

We have used L62 mice as a transgenic model of synucleinopathy with behavioural phenotypes reminiscent of PD, where aggregation was achieved by the overexpression of full-length human α-Syn fused with a membrane-targeting signal sequence peptide and overexpressed under the *Thy-1* promoter^15–17^. For a more in-depth characterisation of L62, we here applied immunohistochemical and biochemical methods to examine the cellular/global deposition pattern and the solubility of α-Syn in these mice. We report neuronal and synaptic α-Syn inclusions in the brain of L62, with an age-preserved distribution pattern across the brain emphasizing a selective vulnerability of neurones to α-Syn inclusions. Furthermore, the presence of multiple α-Syn pools of varying molecular weights that express differential resistance towards solubilisation with Triton, urea and SDS, likely represent oligomers of differently sized configurations.

## Results

L62 mice were used as a model of synucleinopathy. As previously reported, these mice show α-Syn immunoreactivity in different neuronal populations^15,17,18^. The experiments performed in this study were aimed at characterising L62 by (i) establishing specific antibodies able to distinguish between mouse and human α-Syn, (ii) determining the deposition pattern throughout the brain and (iii) determining the solubility properties of α-Syn pools in these mice.

### Specificity of α-Syn antibodies in L62 mice (Experiment I A&B)

Due to the high sequence homology between human and mouse α-Syn, we first examined multiple antibodies for their specificity in immunohistochemistry and immunoblotting. These included the following commercial antibodies: syn204, Clone 42, mAb 211, 3H2897, C20 and 4D6, LB509, as well as TauRx proprietary antibodies mAbs 559, 736 and 874.

**Figure 1:**
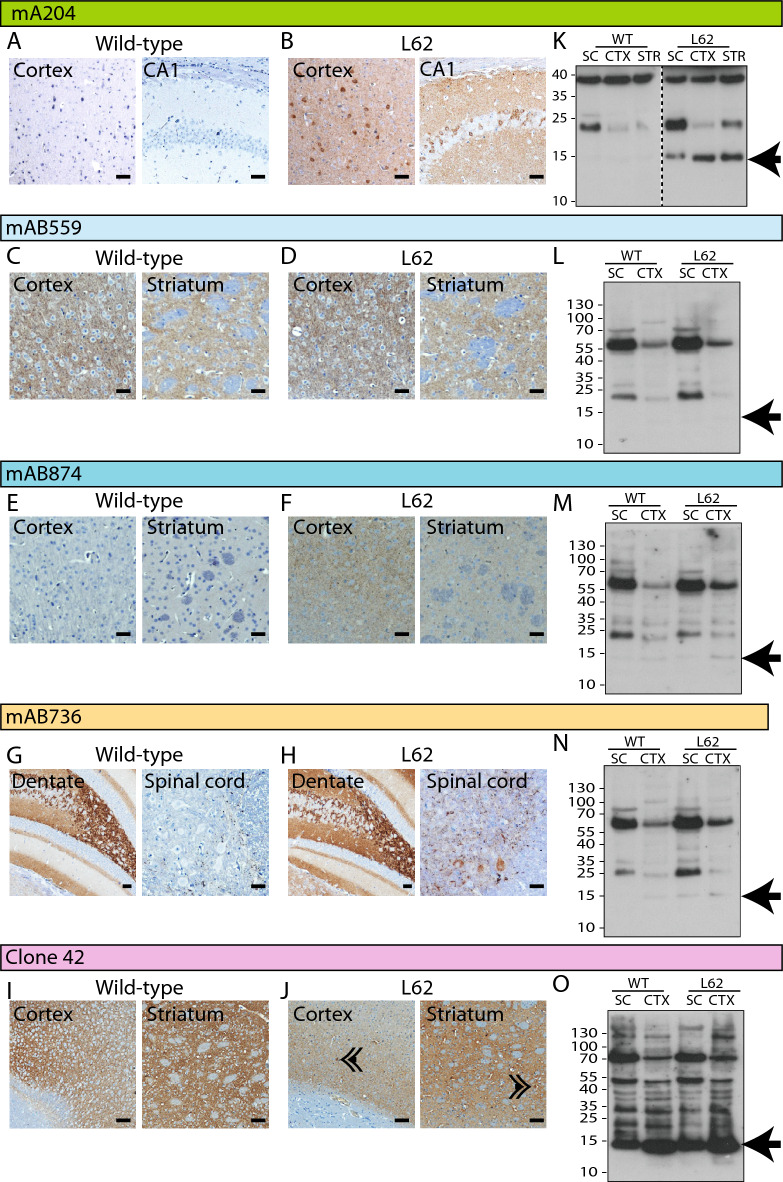
Specificity of anti-α-Syn antibodies in 6-month-old L62 mice (Experiment I A&B). For immunohistochemistry (**A**–**J, 100 µm scale bars**), brain and spinal cord sections from 3 wild-type and 3 L62 mice were analysed (n total = 6 for IHC). Sections were labelled using the five different anti-α-Syn antibodies: syn204 (**A**,**B**), mAb 559 (**C**,**D**), mAb 874 (**E**,**F**), mAb 736 (**G**,**H**), and Clone 42 (**I**,**J**). For immunoblots (**K**–**O**), proteins from brain and spinal cord from 2 wild-type and 3 L62 mice (n total = 5 for IB) were extracted using urea and separated by SDS-PAGE. Two wild-type and 3 L62 mice were analysed. Membranes were labelled using the anti-α-Syn antibodies syn204 (**K**), mAb 559 (**L**), mAb 874 (**M**), mAb 736 (**N**) and Clone 42 (**O**). Arrows indicates α-Syn monomer labelling at ~ 15 kDa. Arrowheads in (**J**) show sporadic neuronal labelling with clone 42.

During immunohistochemical examination, the syn204 did not show any cross reactivity with wild-type mice in any brain region (Fig. 1A), while it showed widespread and prominent cytoplasmic and synaptic staining in L62 (e.g., in cortex and hippocampal CA1 (Fig. 1B)). The cytoplasmic staining was evident as labelled cell bodies, while synaptic α-Syn was seen as small brown puncta in the microscopic images. Antibody mAb 559 showed only synaptic labelling but did not differentiate between genotypes in terms of location or intensity of staining (Fig. 1C,D). Antibody mAb 874 also showed only synaptic labelling, but exclusively in L62 (Fig. 1E,F). The fourth antibody, mAb 736, yielded similar binding in wild-type and L62 brains, but labelled only spinal cord neurones in L62 (Fig. 1G,H). Clone 42 stained synaptic α-Syn, both in wild-type and L62 mice, for example in striatum/cortex, and additionally in a few neurones in L62 brains (Fig. 1I,J). Overall, the cytoplasmic labelling with Clone 42 in L62 was faint and somatic labelling was sparse. LB509 showed cytoplasmic and synaptic staining in L62; some synaptic labelling was also observed in wild-type tissue (Supplementary Fig. 1A).

During immunoblotting, syn204 specifically labelled α-Syn monomers at ~ 15 kDa in L62 but not wild-type mice (see arrow, Fig. 1K), and these α-Syn species are most likely human α-Syn. MAb 559 showed no binding at all at ~ 15 kDa (see arrow, Fig. 1L). MAbs 874 and 736 yielded very faint labelling of α-Syn at ~ 15 kDa; the signal however was similar between genotypes and is likely to be mouse α-Syn (see arrow, Fig. 1M,N). Clone 42 also labelled α-Syn at ~ 15 kDa, but the intensity was stronger in L62 compared to wild-type mice suggesting that this antibody is non-discriminatory between human and mouse α-Syn (see arrow, Fig. 1O). LB509 failed to reveal any signal specific to L62 brains (Supplementary Fig. 1B, see arrow). The monoclonal antibody mAb 211 was like syn204 and specifically revealed human α-Syn monomers at ~ 15 kDa; the signal emerged in L62 but not in wild-type mice (Supplementary Fig. 2A, see arrow). Both, monoclonal antibody 3H2897 and polyclonal antibody C20, labelled only mouse α-Syn at ~ 15 kDa at similar intensity in both genotypes (see arrow, Supplementary Fig. 2B,C). The monoclonal antibody 4D6 resembled Clone 42 and it too labelled both human and mouse α-Syn at ~ 15 kDa (Supplementary Fig. 2D, see arrow). Noteworthy is that all antibodies additionally labelled multiple higher molecular weight bands which were similar between genotypes and are presumably oligomers of syn (human and/or mouse).

In summary, the antibodies tested showed significant cross reactivity between human and mouse α-Syn by immunohistochemistry. All antibodies labelled multiple higher molecular-weight non-specific bands, and none showed exclusive binding to human α-Syn monomers on immunoblots. The only antibody that robustly distinguished between wild-type and L62 genotypes was syn204, that labelled α-Syn at ~ 15 kDa in tissues from L62 but not wild-type mice. In addition, syn204 was specific and discriminatory for L62 during IHC. For consistency, syn204 was therefore used for all of the following experiments.

### Deposition pattern of α-Syn in L62 (Experiment II)

**Figure 2:**
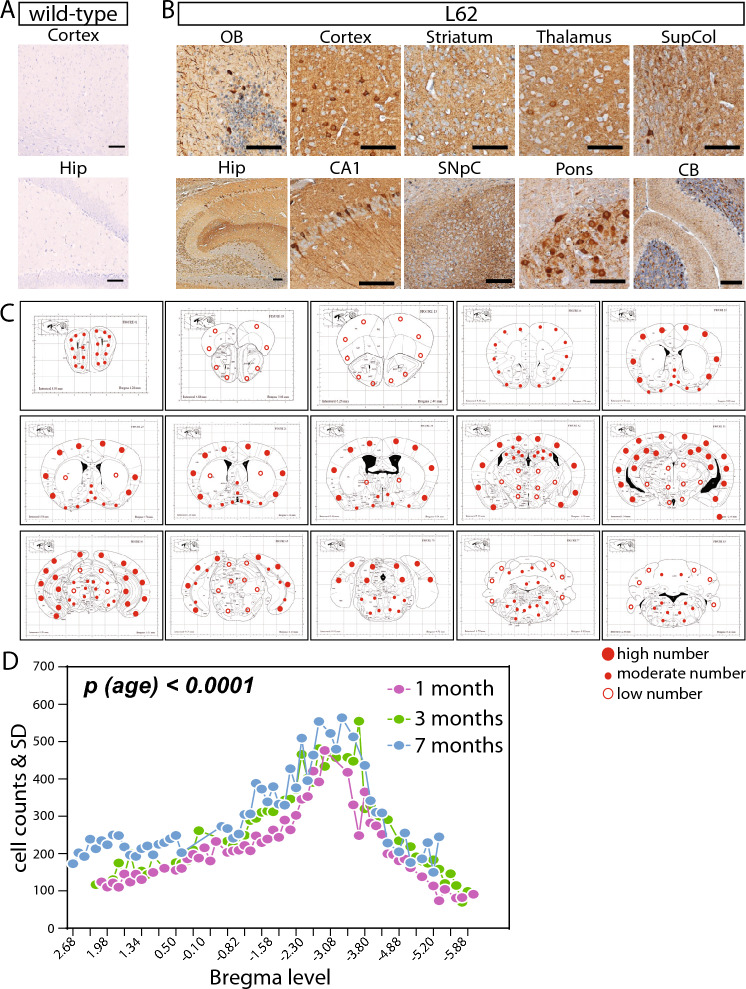
Expression pattern and quantification of α-Syn in L62 brains (Experiment II). Representative images of α-Syn immunoreactivity using syn204 (100 µm scale bars) showed no immunoreactivity in wild-type mice (**A**) but prominent staining in multiple regions in L62 (**B**). Region-specific semi-quantification of α-Syn-positive neurones showed greatest staining in cortex and hippocampus (**C**; large dots for high cell counts, small dots for moderate cell counts, and open dots for low cell counts). Additionally, quantitative values from whole sections are plotted as a function of brain levels and shown as group mean (**D**). Mice were aged 1, 3 or 7 months (2 wild-type and 4 L62 mice were analysed for each age; n total = 18). OB: olfactory bulb, SupCol: superior colliculus, Hip: hippocampus, SnpC: substantia nigra pars compacta, CB: cerebellum.

Having established the presence of human α-Syn immunoreactivity in L62 and its absence in wild-type mice with syn204 (Fig. 1A,B), we next investigated the deposition pattern and density of α-Syn throughout the brain. L62 mice showed considerable α-Syn immunoreactivity in multiple areas, e.g., cortex, striatum, thalamus, CA1, pons and cerebellum, while wild-type mice did not show any immunoreactivity in any of these regions (Fig. 2A,B). Semi-quantitation in L62 yielded high α-Syn deposition levels in all areas of cortex, hippocampus, and pons; moderate levels in the basal forebrain, olfactory bulb, and superior colliculus; only a few cells with α-Syn inclusions were seen in striatum, substantia nigra, thalamus, and cerebellum (Fig. 2C, large dots for high cell counts, small dots for moderate cell counts, and open dots for low cell counts). Quantification of these α-Syn-positive neurones along the longitudinal axis of the brain as a function of distance from Bregma revealed significant differences depending on brain level (Fig. 2D, 2-way ANOVA effect of brain level p < 0.0001). For example, the greatest α-Syn cell counts were found in midbrain regions (Bregma − 1.58 to − 4.34), while very low α-Syn positive cells were counted in forebrain (Bregma > 0.5) and hindbrain (Bregma < − 5.0). A similar deposition pattern of α-Syn across L62 age groups was observed (Fig. 2D), but nonetheless, there was a significant increase in α-Syn cell counts with age in L62 (2-way ANOVA effect of age p < 0.0001).

In summary, widespread α-Syn labelling was evident in L62 brains and was greatest in cortex and hippocampus. The deposition of α-Syn in the brains of L62 mice increased progressively with age but the pattern of deposition was preserved across age groups.

### Solubility of α-Syn in L62 (Experiment III)

Based on the accumulation of human α-Syn in L62 brains, we applied biochemical fractionations to further characterise the α-Syn species identified with syn204.

**Figure 3:**
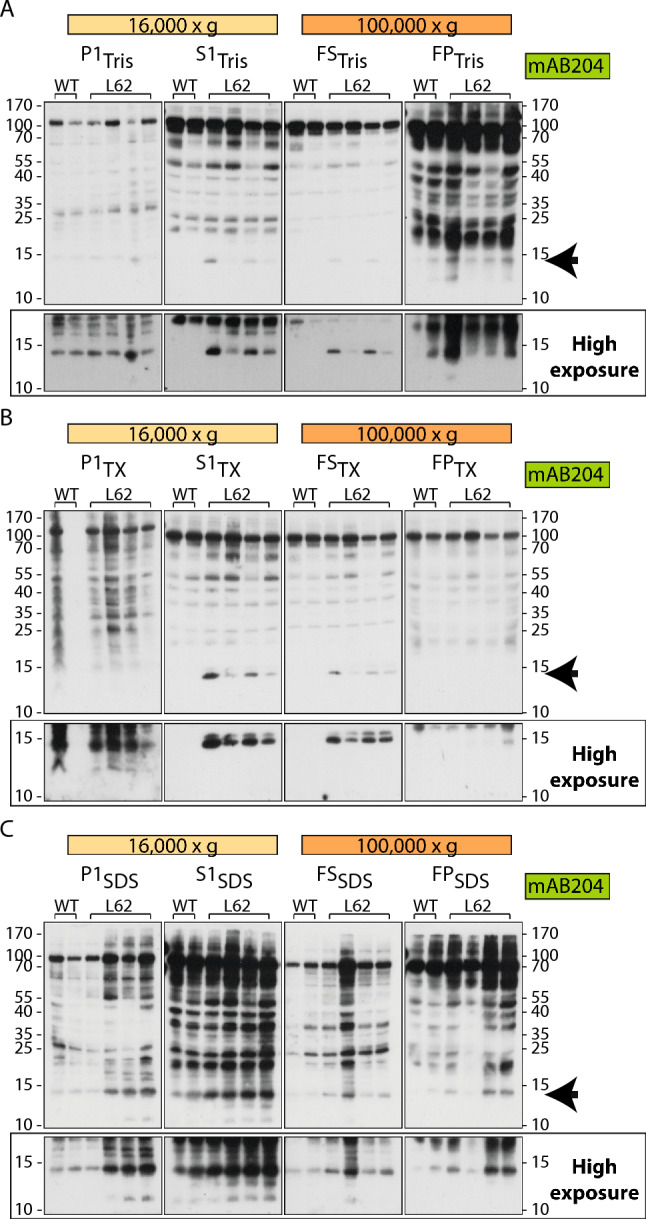
Solubility of α-Syn from L62 brains using sequential supernatant fractionation (Experiment III). Proteins from L62 brain tissue were extracted using different buffers in combination with low- (16,000×*g*) and high-speed (100,000×*g*) centrifugation and separated by SDS-PAGE. (**A**) For the Tris extraction (protocol 2), low- and high-speed fractions were generated by incubation in Tris buffer. The resulting fractions were P1_Tris_, S1_Tris_, FS_Tris_ and FP_Tris_. (**B**) For the mixed-speed sequential Tris-TX extraction (protocol 3), low- and high-speed fractions were generated by incubation in Tris-Triton buffer. The resulting fractions were P1_TX_, S1_TX_, FS_TX_ and FP_TX_. (**C**) For the mixed-speed sequential Tris-SDS extraction (protocol 4), low- and high-speed fractions were generated by incubation in Tris-SDS buffer. The resulting fractions were P1_SDS_, S1_SDS_, FS_SDS_) and FP_SDS_. Proteins were transferred to PVDF membranes and immunoblots were developed using syn204. Mice were 6-months old with 2 wild-type and 4 L62 mice analysed (n total = 6). Arrows indicate position of α-Syn monomer labelling at ~ 15 kDa. The lower part of the immunoblots covering ~ 10–20 kDa are also shown with increased exposure.

The first set of experiments included detergent-free sequential supernatant fractionation. Using the detergent-free approach with Tris, in the first pellet P1_Tris_, a monomeric ~ 15 kDa signal was detected in both wild-type and L62. This most likely represents membrane-bound murine α-Syn. In the low-speed Tris-soluble supernatant S1_Tris_, a L62 specific signal was seen at ~ 15 kDa and most likely represents human α-Syn. When this supernatant was fractionated further, only small amounts of human α-Syn were transferred into the high-speed supernatant FS_Tris_. The majority however, remained in the high-speed pellet FP_Tris_. Nonetheless, some binding was also seen in wild-type samples in FP_Tris_ at ~ 15 kDa but it was much weaker than in L62 (Fig. 3A, see arrow). When the same procedure was carried out in the presence of Triton, similar amounts of human α-Syn were detected at ~ 15 kDa in the low-speed supernatant S1_TX_, while it was entirely depleted from FP_TX_ and transferred into FS_TX_ through the addition of Triton (Fig. 3B, see arrow). The transgenic α-Syn pool that was sedimented in these conditions is therefore in the form of oligomeric complexes of molecular weight of up to 500 kDa, since TX would disrupt any membrane complexes (vesicles.niifhm.ru for particle size estimation in nm; https://sedgeochem.uni-bremen.de/dalton.html for size conversion from nm to Da). Noteworthy are bands of molecular weights ranging between 20 and 70 kDa, especially in S1_Tris_ and S1_TX_ fractions, with some labelling in wild-type but strong labelling in L62, that could represent α-Syn dimers/oligomers (Fig. 3A,B).

When SDS was applied, α-Syn at ~ 15 kDa was evident in wild-type and L62 samples, although the signal was considerably stronger for L62 mice (Fig. 3C, see arrow). We assume that these pools contain both mouse and human α-Syn that share the same (partial) resistance towards SDS. These α-Syn pools are equally distributed between supernatants and pellets, but more has accumulated in the low than the high-speed fractions. They are therefore most likely oligomeric complexes of varying molecular weights ranging from 200 to 500 kDa (100,000 and 16,000×*g*, respectively, see above for particle size estimation) that have partial resistance to SDS solubilisation, as some remains in the pellet.

**Figure 4:**
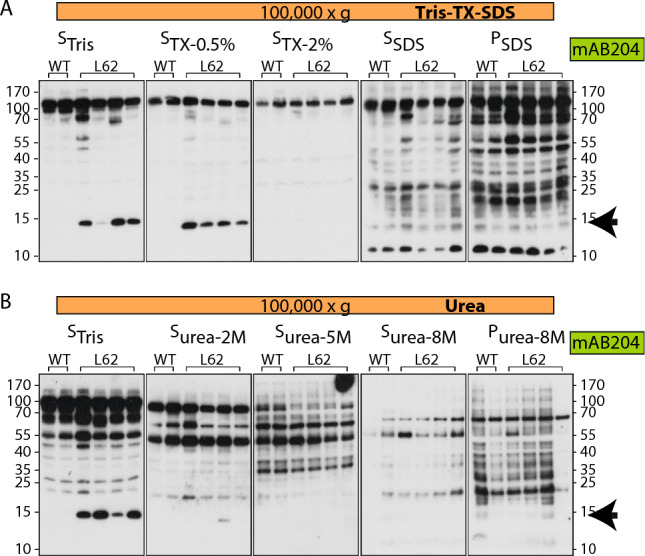
Solubility of α-Syn from L62 brains using sequential pellet fractionation (Experiment III). Proteins from L62 brain tissue were extracted using different buffers and separated by SDS-PAGE. (**A**) For the high-speed Tris-Triton-SDS extraction (protocol 5), tissue was incubated sequentially in Tris, Tris-Triton and Tris-SDS and centrifuged at 100,000×*g*. The resulting fractions were S_Tris_, S_TX-0.5%_, S_Tx-2%_, S_SDS_ and P_SDS_. (**B**) For the high-speed urea extraction (protocol 6), tissue was incubated in increasing concentrations of urea and centrifuged at 100,000×*g*. The resulting fractions were S_Tris_, S_urea-2 M_, S_urea-5 M_, S_urea-8 M_ and P_urea-8 M_. Membranes were labelled using syn204. Extraction was conducted using the same mice as in Fig. 3. Arrows indicate position of α-Syn monomer labelling at ~ 15 kDa.

When applying the sequential supernatant fractionation using Tris, we found that the majority of human α-Syn remained in the high-speed pellet FP_Tris_ (Fig. 3A, right panel). Therefore, a second set of experiments was conducted where the pellet was treated with buffers of increasing stringency (Triton-SDS or urea). In supernatant S_Tris_, a L62 specific signal was seen at ~ 15 kDa and represents human α-Syn, confirming the above findings. More of the human α-Syn was released when 0.5% Triton was added to the pellet to obtain S_TX-0.5%_, while a further increase of Triton from 0.5 to 2% in the subsequent pellet failed at solubilising more of it into S_TX-2%_ (Fig. 4A, see arrow). The addition of SDS to the penultimate pellet released a further pool of monomeric α-Syn at ~ 15 kDa, which was detected in both wild-type and L62 samples, possibly representing mouse α-Syn (Fig. 4A, fractions S_SDS_ and P_SDS_). When urea was applied instead of Triton-SDS, no additional α-Syn pools were solubilised, but all of the L62-specific human α-Syn was found in the first supernatant S_Tris_ (Fig. 4B, see arrow).

In summary, different pools of α-Syn exist in L62 brains, with Triton-resistant oligomeric pools exclusively found for human α-Syn in L62, and SDS-soluble pools containing human and mouse α-Syn of similar biochemical properties.

## Discussion

Aggregation of human α-Syn in L62 mice was achieved by the overexpression of a full-length construct fused with a membrane-targeting signal sequence peptide and overexpressed under the *Thy-1* promoter. We have used this expression cassette before in a model expressing a truncated species of tau and introduced a membrane-targeting sequence to direct the expressed protein to the endoplasmic reticulum membrane. This offered benefits by ameliorating the toxicity of free protein and by providing a seed on which to induce template-directed aggregation of tau within neurons^19^. This was also applied in the generation of L62, since a corresponding cellular model has confirmed the aggregation of such a fusion protein^16^. This novel approach in the creation of α-Syn models led to the neuronal expression of α-Syn reminiscent of synucleinopathies. Expression of human α-Syn is widespread and cortical and hippocampal regions are particularly dense. Novel findings reported here include a preserved α-Syn distribution pattern across L62 age groups. Alpha-Syn is greatest at mid-brain levels (cortex and hippocampus), but also evident in basal forebrain and hindbrain regions. In addition, we present evidence for the existence of different pools of α-Syn in L62 brains that form oligomeric complexes of varying molecular weights, with Triton-resistant pools exclusively found for human α-Syn but not murine α-Syn.

The link between α-Syn mutations and deposition of α-Syn in PD brains has encouraged the generation of several lines of α-Syn transgenic mice over the last couple of decades^11,20,21^, with several models expressing α-Syn with and without pathogenic mutations under the control of different promotor systems, e.g. the prion protein or the neuron-specific *Thy-1* promoter. Early work has emphasized that mutations in the α-Syn gene are not required or sufficient to induce protein aggregation^22,23^. For example, transgenic mice overexpressing A30P mutant α-Syn under the prion protein promoter accumulated α-Syn in many regions of the brain but with no evidence of aggregation^24,25^. In double-transgenic mice overexpressing A30P and A53T α-Syn in dopaminergic neurones under the tyrosine hydroxylase promoter, inclusions were evident in cell bodies in ventral tegmental area/substantia nigra as well as in terminals of the striatum and the nucleus accumbens^26^. However, even high-expressing mouse lines were devoid of α-Syn aggregation^27^. On the other hand, filamentous and pathogenic α-Syn aggregates were seen in both, wild-type and A53T α-Syn transgenic mice^28,29^. Indeed, in L62 mice, which overexpress wild-type human α-Syn targeted to membranes, the transgene product induces motor impairment, confirming its pathogenicity^16^.

In L62, α-Syn inclusions are predominant in the cortex and the hippocampus, but low to moderate in the basal forebrain, striatum, substantia nigra, and the thalamus. This pattern seems to be like a mixture of PD and DLB pathologies, where inclusions occur in subcortical neurones and in cortical brain areas, respectively^9–12^. Certainly, differences in the deposition pattern between α-Syn transgenic mouse lines are driven, at least in part, by the different promoters used to achieve overexpression. For example, dopaminergic expression is achieved via the tyrosine hydroxylase promoter^26^, cortical and subcortical expression via the *Thy-1* promoter^15,30^, while α-Syn expression seems to be restricted to the hippocampus when overexpressed under the platelet-derived growth factor B-chain (PDGF-B) element^23,30^. On the other hand, we have observed an age-preserved distribution pattern across the brain in L62 emphasizing a selective vulnerability of neurones to α-Syn inclusions, and it has been proposed that this selective vulnerability, both in terms of brain regions and neuronal populations, is closely related to endogenous (murine) α-Syn expression^31,32^. In L62 brains, human α-Syn can be revealed in glutamatergic and cholinergic neurones and synapses^15,17,18^, and it has been reported that endogenous α-Syn is uniformly expressed in glutamatergic synapses, and has a broadly similar expression in excitatory synapses overall^31^. This selective vulnerability is reflected by the unique neuropathological Braak staging in the different forms of synucleinopathies^32^. The selective vulnerability is likely to drive the disease specific α-Syn deposition pattern and possibly the transmission and spread of α-Syn^33^. We hypothesize that cell type specific environmental conditions facilitate/inhibit the formation of these different α-Syn species. Consequently, the large, as well as intermediate-sized oligomers were identified in both distinct and overlapping fractions, which emphasize the pathogenic nature of the α-Syn species, and also explain why the pathology in L62 seems to reflect a mixture of both PD and DLB.

The α-Syn species in L62 were in part resistant to both Triton and SDS, confirming early findings reported for wild-type and A53T α-Syn transgenic mice, where progressively more α-Syn monomers were solubilised in buffers of increasing stringency^29^. Moreover, the appearance of α-Syn species of different molecular mass, which are only visible following detergent solubilisation and unique to L62 brains, can directly link these species to neuropathology^24^.

The most comparable model to our L62 is the line 61 model^23,30^. While both models express α-Syn without pathogenic mutations under the control of the neuron-specific *Thy-1* promoter, there are also obvious differences such as the background mouse stains (C57BL6J in L62 but C57BL6J/DBA2 in line 61) and the membrane-targeting element in L62. Different forms of aggregated α-Syn were also reported in line 61 using different solubilisation buffers, but their use of ELISA assays makes a direct comparison with our findings impossible^34^.

In humans, the equilibrium between the different α-Syn species seems to be abnormal in synucleinopathies, and it has been shown that the α-Syn dimer/monomer ratio is increased in PD and DLB brain^35^. However, recent findings suggest that the ratio between aggregated and monomeric α-Syn species is decreased in PD and DLB brains^33^.

In L62, different pools of α-Syn complexes of varying molecular weights were identified. However, a precise quantification of these α-Syn complexes depends on the availability of antibodies for selective immunoprecipitation to generate purified fractions. As shown here, the significant antibody cross-reactivity between species (mouse and human) and the presence of multiple non-specific bands during immunolabelling impedes this task. Our own mAbs 559, 736 and 874 and the commercial Clone 42 antibody specifically labelled α-Syn in differentiated DH60.21 cells^16^, but failed to do so in L62 tissue despite the fact that both carry the same construct. This is likely due to specific events (cell culture vs. mouse) which influence α-Syn conformations.

In summary, neuronal and synaptic α-Syn inclusions in L62 brains were evident and widespread in cortical and subcortical regions. The age-preserved distribution pattern across the brain emphasizes a selective vulnerability of neurones to α-Syn inclusions. We here speculate that this distribution pattern is directly related to the presence of multiple α-Syn pools/species of varying molecular weights and biochemical solubilities, and their further characterisation could be crucial for the development of novel disease-modifying treatments.

## Methods

### Animals and study design

All animal experiments were performed in accordance with the European Communities Council Directive (63/2010/EU), approved by the German Animal Research Ethics Committee of LAGESO (Experiments I–III), and comply with the ARRIVE guidelines 2.0^36^. We have used L62 mice as a model of synucleinopathy. These mice overexpress full-length human α-Syn fused with a signal sequence peptide under control of the *Thy-1* promoter and L62 have widespread human α-Syn immunoreactivity^15,16^. Male homozygous L62 and C57BL6/J wild-type litters were bred commercially in individually ventilated cages (Forschungseinrichtung fuer Experimentelle Medizin (FEM), Berlin, Germany) and delivered to the experimental facilities 1 week prior to tissue harvest. They were housed by genotype in small colonies up to six mice in open housing Macrolon type III cages, which were enriched with corncob bedding, paper strips and cardboard tubes. The temperature (20–22 °C) and humidity (60–65%) in the holding rooms were kept constant. The air exchange rate was set to 17–20 changes per hour and the day-night cycle to 12 h (lights on at 6 a.m.). Mice had free access to food and water and were 1, 3 or 6–7 months of age when tissue was harvested. A total of 35 mice were used in this study (see Table 1).

**Table 1:** Study details.

Exp	Study type (antibody)	Method	Age (months)	Sex	Number of mice	Tissue
I-A	Antibody specificity	IHC	6	Male	6 (3 WT and 3 L62)	Fore- and midbrain/SC
I-B		IB	6	Male	5 (2 WT and 3 L62)	Cortex/striatum/SC
II	α-Syn quantification (syn204)	IHC	1	Male	6 (2 WT and 4 L62)	Whole brain
			3		6 (2 WT and 4 L62)	
			7		6 (2 WT and 4 L62)	
III	α-Syn solubility (syn204)	IB	6	Male	6 (2 WT and 4 L62)	Cortex

*Exp*. experiment, *IHC* immunohistochemistry, *IB* immunoblotting, *WT* wild-type mice, *L62* α-Syn transgenic mice, *SC* spinal cord.

### Animal sacrifice, wax-embedding of brains, and tissue sectioning for immunohistochemistry

For antibody specificity studies (Experiment I-A), 3 wild-type and 3 L62 mice (6 months, Table 1) were used. For quantitative α-Syn studies with syn204 (Experiment II), 6 wild-type and 12 L62 mice were used (1, 3 or 7 months of age, Table 1). Mice were sacrificed by cervical dislocation. The top of the skull was exposed, and the overlying bone plates removed to allow harvest of the brain. Right brain hemispheres were dissected, fixed overnight at room temperature (RT) in 10% (v/v) neutral-buffered formalin and embedded in paraffin. Hemi-brains were coronally sectioned at 5 µm using a rotary microtome (HM 325, Leica Biosystems), and mounted onto SuperFrost™ glass slides (Thermo Fisher Scientific, three sections per slide). Fore- and mid-brain sections were collected and labelled with different anti α-Syn antibodies (Table 2). For age-related quantitative histology with syn204, each 10th section was collected across the whole brain according to the mouse brain atlas^37^, resulting in 55 glass slides (with 3 sections each) per mouse. These sections (2970 in total) were used to quantify transgenic α-Syn in entire brains.

**Table 2:** List of primary α-Syn antibodies.

Antibodies	Source	Antibody-ID	Host	Immunogen	Dilution IHC	Dilution IB
syn204	SCB	sc-32280	Mouse	Recombinant human α-syn	1:100	1:100
mAb 559	TauRx	F-Syn7, clone 559	Mouse	Recombinant full-length human α-syn	1:100	1:100
mAb 874	TauRx	F-Syn7, clone 874	Mouse	Recombinant full-length human α-syn	1:100	1:100
mAb 736	TauRx	T-Syn8, clone 736	Mouse	Recombinant truncated human α-syn (aa. 91–326)	1:100	1:100
Clone 42	BDB	15895639	Mouse	Truncated rat synuclein-1 (aa. 15–123)	1:100	1:500
mAb 211	SCB	sc-12767	Mouse	Recombinant truncated human α-syn (aa. 121–125)	N/A	1:100
LB509	Abcam	Ab27766	Mouse	α-syn (aa. 115–122)	1:500	1:500
3H2897	SCB	sc-69977	Mouse	Recombinant human α-syn	N/A	1:500
C20	SCB	sc-7011R	Rabbit	C-terminus of human α-syn	N/A	1:500
4D6	Abcam	Ab1903	Mouse	Recombinant human α-syn	N/A	1:500

*BDB* BD Biosciences, *mAb* monoclonal antibody, *N/A* not applicable, *SCB* Santa Cruz Biotechnology. All primary antibodies are monoclonal, apart from C20 which is polyclonal.

### Immunohistochemistry and manual counting of α-Syn positive neurones in brain

Antibody specificity studies (syn204, mAb 559, mAb 874, mAb 736, Clone 42; Experiment I-A) were performed on sections from wild-type and L62 mice stained together in one immunohistochemistry staining box; one box was used for each antibody. For quantitative α-Syn studies using syn204 (Experiment II), whole brain section series (55 sections per mouse) were randomized over immunohistochemistry staining boxes in a balanced manner so that each box contained all age groups and genotypes. Sections were deparaffinised and boiled in 10 mM citric buffer (pH 6.0), incubated in 0.3% (v/v) hydrogen peroxidase solution, and blocked in blocking solution (0.1% (w/v) bovine serum albumin in phosphate buffered saline, pH 7.4) for 20 min. Sections were incubated in primary antibody (1 h at RT), then in biotinylated goat anti-mouse IgG secondary antibody (Dako Denmark, Glostrup, Denmark, 1 h at RT) and finally in diaminobenzidine solution (Dako Denmark). Primary and secondary antibodies were diluted in blocking solution. Sections were embedded in Neo-Mount (Merck Millipore, Burlington, MA, USA), and images were taken using a light microscope (Axio imager M1, Carl Zeiss, Jena, Germany, equipped with a camera and computer) and saved as JPEG.

For antibody specificity studies using the different anti α-Syn antibodies (Experiment I-A), images were taken from fore- and midbrains at 100× magnification and assessed semi-quantitatively in areas of interest. Areas with high cell counts (α-Syn positive neurones in more than 40% of the area) were scored with large dots, those with moderate cell counts (more than 20%) scored with small dots, and those with low cell counts (less than 10%) scored with open dots.

Images for age-related quantitative analyses with syn204 (Experiment II) were captured using the MosaiX function of the AxioVision software (version 4.1, Carl Zeiss). This specific software tool scans the entire surface of a section and acquires multiple individual images, which are then combined to form a single image that covers the complete brain hemisphere. Alpha-Syn positive neurones were counted in these images manually and values spanning the entire brain were plotted as a function of Bregma level^37^. Antibody details and dilutions are given in Table 2.

### Animal sacrifice, urea protein extraction and antibody specificity studies (protocol 1)

Mice were 6 months old when sacrificed by cervical dislocation (2 wild-type and 3 L62 mice, Table 1; Experiment I-B). The top of the skull was exposed, and the overlying bone plates removed to allow harvest of the whole brain. The cortex and the striatum were dissected, transferred into separate 1.5 mL reaction tubes, immediately frozen in liquid nitrogen, and kept at − 80 °C until use. Additionally, spinal cord tissue was dissected, snap frozen and stored at − 80 °C. Tissue from each mouse was crushed and used for urea extraction according to protocol 1 as follows. Crushed brain tissue was incubated for 45 min at RT in six volumes of urea extraction buffer (7 M urea, 2 M thiourea, 2% ampholyte 2–4, 70 mM DTT, 25 mM Tris/HCl pH 8.0, 50 mM KCl, 3 mM EDTA, 2.9 mM benzamidine and 2.1 µM leupeptin) and separated by centrifugation at 16,000×*g* for 45 min at RT. The supernatant was transferred to new tubes and used for electrophoresis. The protein concentration in the supernatants was determined using the Bradford Reagent (Carl Roth, Karlsruhe, Germany) according to the manufacturer´s recommendations. The urea supernatants were used for antibody specificity studies (Experiment I-B), and blots were labelled using the monoclonal anti α-Syn antibodies syn204, mAbs 559, 874 and 736, Clone 42, mAb 211, 3H2897, C20 and Ab1903 (see Table 2 for details).

### Animal sacrifice, sequential protein extraction and solubility studies (protocols 2–6)

**Figure 5:**
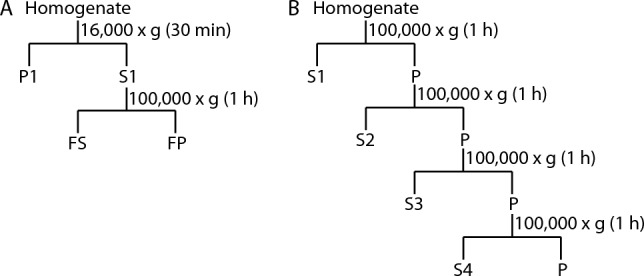
Extraction protocols for sequential fractionation. Sequential fractionation of brain tissue homogenate was applied at indicated centrifugation speeds to allow separation and enrichment of different protein pools. (**A**) For supernatant fractionation, Tris (protocol 2), Tris-Triton (protocol 3) or Tris-Triton-SDS (protocol 4) were used for protein extraction. (**B**) For pellet fractionation, Tris-Triton-SDS (protocol 5) or Tris-urea (protocol 6) were used.

Mice aged 6 months were sacrificed by cervical dislocation (2 wild-type and 4 L62 mice, Table 1; Experiment III). After removal of the entire brain (details, see above) the cortex was dissected, transferred into 1.5 mL reaction tubes, immediately frozen in liquid nitrogen, and kept at − 80 °C until use. Tissue from each individual mouse was crushed, divided into five aliquots, and each aliquot was used for α-Syn solubility studies applying the extraction protocols described below (protocols 2–6; Fig. 5).

Protocol 2 (Fig. 5A). For the sequential supernatant fractionation using Tris, crushed frozen brain tissue was incubated for 30 min in 5 volumes Tris buffer (30 mM Tris containing complete protease inhibitor cocktail tablets) at 4 °C, and the homogenate centrifuged for 30 min at 16,000×*g* and 4 °C. The pellet (P1_Tris_) was suspended in 450 µL Tris buffer, 50 µL of the supernatant (S1_Tris_) were retained and the remaining supernatant centrifuged for 1 h at 100,000×*g* and 4 °C. The resultant high-speed supernatant (FS_Tris_) was retained. The pellet (FP_Tris_) was suspended in 1 volume of Tris buffer and retained. The resulting fractions were T_Tris_, P1_Tris_, S1_Tris_, FS_Tris_ and FP_Tris_.

Protocol 3 (Fig. 5A). For the sequential supernatant fractionation using Tris-Triton, the same procedure as described in protocol 2 was used except for the Tris buffer being exchanged for Tris-Triton (0.1% v/v Triton). The resulting fractions were T_TX_, P1_TX_, S1_TX_, FS_TX_ and FP_TX_.

Protocol 4 (Fig. 5A). For sequential supernatant fractionation using SDS, protocol 2 was followed except for the Tris buffer being exchanged for Tris-SDS (0.5% w/v SDS). The resulting fractions were T_SDS_, P1_SDS_, S1_SDS_, FS_SDS_ and FP_SDS_.

Protocol 5 (Fig. 5B). For high-speed sequential pellet fractionation using Tris-Triton-SDS, crushed frozen brain tissue was incubated for 1 h in 5 volumes Tris buffer (10 mM Tris containing complete protease inhibitor cocktail tablets) at 4 °C, and the homogenate centrifuged at 4 °C and 100,000×*g* for 1 h. The resultant first supernatant was retained (= S_Tris_) and the pellet suspended in 450 µL Tris-Triton X-100 (0.5% v/v Triton), incubated for 1 h at 4 °C and centrifuged for 1 h at 4 °C and 100,000×*g*. The second supernatant S_TX-0.5%_ was retained and the pellet suspended in 450 µL Triton X-100 (2% v/v Triton), incubated for 1 h and 4 °C and centrifuged for 1 h at 4 °C and 100,000×*g*. The third supernatant S_TX-2%_ was retained and the pellet suspended in 450 µL Tris-SDS (0.5% w/v SDS), incubated for 1 h at 12 °C and centrifuged at 12 °C and 100,000×*g*. The fourth supernatant S_SDS_ was retained, and the final pellet (P_SDS_) suspended in 1 volume Tris-SDS (0.5% w/v SDS). The resulting fractions were S_Tris_, S_TX-0.5%_, S_Tx-2%_, S_SDS_ and P_SDS_.

Protocol 6 (Fig. 5B). For high-speed sequential pellet fractionation using Tris-urea, crushed frozen brain tissue was incubated for 1 h in 5 volumes Tris buffer (60 mM Tris containing complete protease inhibitor cocktail tablets) at 4 °C, and the homogenate centrifuged for 1 h at 4 °C and 100,000×*g*. The resultant first supernatant was retained (= S_Tris_) and the pellet suspended in 450 µL Tris-2 M urea, incubated for 1 h at RT and centrifuged for 1 h at RT and 100,000×*g*. The second supernatant S_urea-2 M_ was retained, and the pellet suspended in 450 µL Tris-5 M urea, incubated at RT for 1 h and centrifuged for 1 h at RT and 100,000×*g*. The third supernatant S_urea-5 M_ was retained, and the pellet suspended in 450 µL Tris-8 M urea, incubated for 1 h at RT and centrifuged for 1 h at RT and 100,000×*g*. The fourth supernatant S_urea-8 M_ was retained, and the final pellet, P_urea-8 M_, suspended in 1 volume Tris-8 M urea. The resulting fractions were S_Tris_, S_urea-2 M_, S_urea-5 M_, S_urea-8 M_ and P_urea-8 M_.

Protein concentrations for all fractions were determined using the Bradford Reagent (Carl Roth, Karlsruhe, Germany) according to the manufacturer´s recommendations. Samples from protocols 2–6 were used for solubility studies and blots were labelled using syn204 (Experiment III).

### Tricine SDS-PAGE, protein transfer and immunolabelling

Samples from all fractions (protocols 1–6) were mixed with Laemmli buffer, boiled at 95 °C for 5 min, and loaded onto Tris-tricine gels (Biorad #4563063) with 25 µg protein per lane. The cathode buffer consisted of 100 mM Tris, 100 mM tricine and 1% (w/v) SDS and the anode buffer contained 100 mM Tris and 0.07% (v/v) HCl. Gels were transferred to PVDF membranes in transfer buffer (300 mM Tris, 6% (v/v) acetic acid, pH 8.6) at 0.4 mA/cm^2^ and 4 °C. Membranes were blocked for 1 h at RT in blocking solution (4% (w/v) BSA in TBS with 0.2% (v/v) Tween-20; TBS-T), incubated overnight at 4 °C in primary antibody, diluted in blocking solution (see Table 2 for dilution details), washed 3 × 10 min in TBS-T and incubated for 1 h at RT in appropriate HRP-conjugated secondary antibody (goat-anti-mouse # P0447 or goat-anti-rabbit # P0448, both from Agilent, Dako) diluted 1:5000 in blocking solution. After washing three more times in TBS-T, membranes were overlaid for 1 min with ECL solution (100 mM Tris pH 8.5, 1.25 mM luminol, 200 µM p-coumaric acid, 0.01% (v/v) H_2_O_2_). The chemiluminescence signals were detected on hyper-films (GE Healthcare, USA), and films scanned and saved as TIFF. Details for antibodies used are given in Table 2. Uncropped membrane images are provided in the Supporting Information (Supplementary Figs. 3–6).

### Data analyses

Immunohistochemistry data are plotted as group mean, and standard deviation (SD). GraphPad Prism software (version 8.00; GraphPad Software Inc.) was used to conduct statistical analyses and to generate graphs. Quantitative α-Syn histopathology data were analysed using 2-way analysis of variance (ANOVA) for age and brain level as main effects and with Bonferroni multiple comparison correction post-hoc test. Differences were considered significant at alpha = 5% (p < 0.05).

### Institutional review board statement

All animal experiments were performed in accordance with the European Communities Council Directive (63/2010/EU), approved by the German Animal Research Ethics Committee of LAGESO, and comply with the ARRIVE guidelines 2.0. No human samples were used in this study.

### Supplementary Information

Supplementary Figures.

## Data Availability

All data are provided within the manuscript or the Supplementary information.
