# Supplementary material for: Solubility of α-synuclein species in the L62 mouse model of synucleinopathy
Source: Sci Rep. 2024 Mar 14;14:6239. doi: 10.1038/s41598-024-56735-6 (PMC10940722; doi:10.1038/s41598-024-56735-6)

Supplementary information

Solubility of α-Synuclein species in the L62 mouse model of synucleinopathy

Karima Schwab[1,2*], Mandy Magbagbeolu[2], Franz Theuring[2], Charles R. Harrington[1,3], Claude M. Wischik[1,3], Gernot Riedel[1]

[1] School of Medicine, Medical Sciences and Nutrition, University of Aberdeen, Forester hill, Aberdeen, AB25 2ZD, UK

[2] Institute of Pharmacology, Charité - Universitätsmedizin Berlin, Hessische Str. 3-4, 10115 Berlin, Germany

[3] TauRx Therapeutics Ltd., 395 King Street, Aberdeen, AB24 5RP, UK

*Corresponding author: Karima Schwab
Email: karima.schwab@abdn.ac.uk

Running title: α-Syn species in L62

Key words: Alpha-synuclein, Parkinson's disease, mouse model, protein aggregation, solubility

Supplementary Figure 1: Specificity of LB509 in L62 mice. Representative immunohistochemistry images for wild-type and L62 mice labelled with the antibody LB509 (A). Immunoblots of sequentially fractionated wild-type (WT) and L62 brain tissue using Tris-Triton-SDS extraction (protocol 5) and labelling with LB509. Arrows indicate lack of L62 human specific α-Syn monomer at the expected molecular weight of ~15kDa.

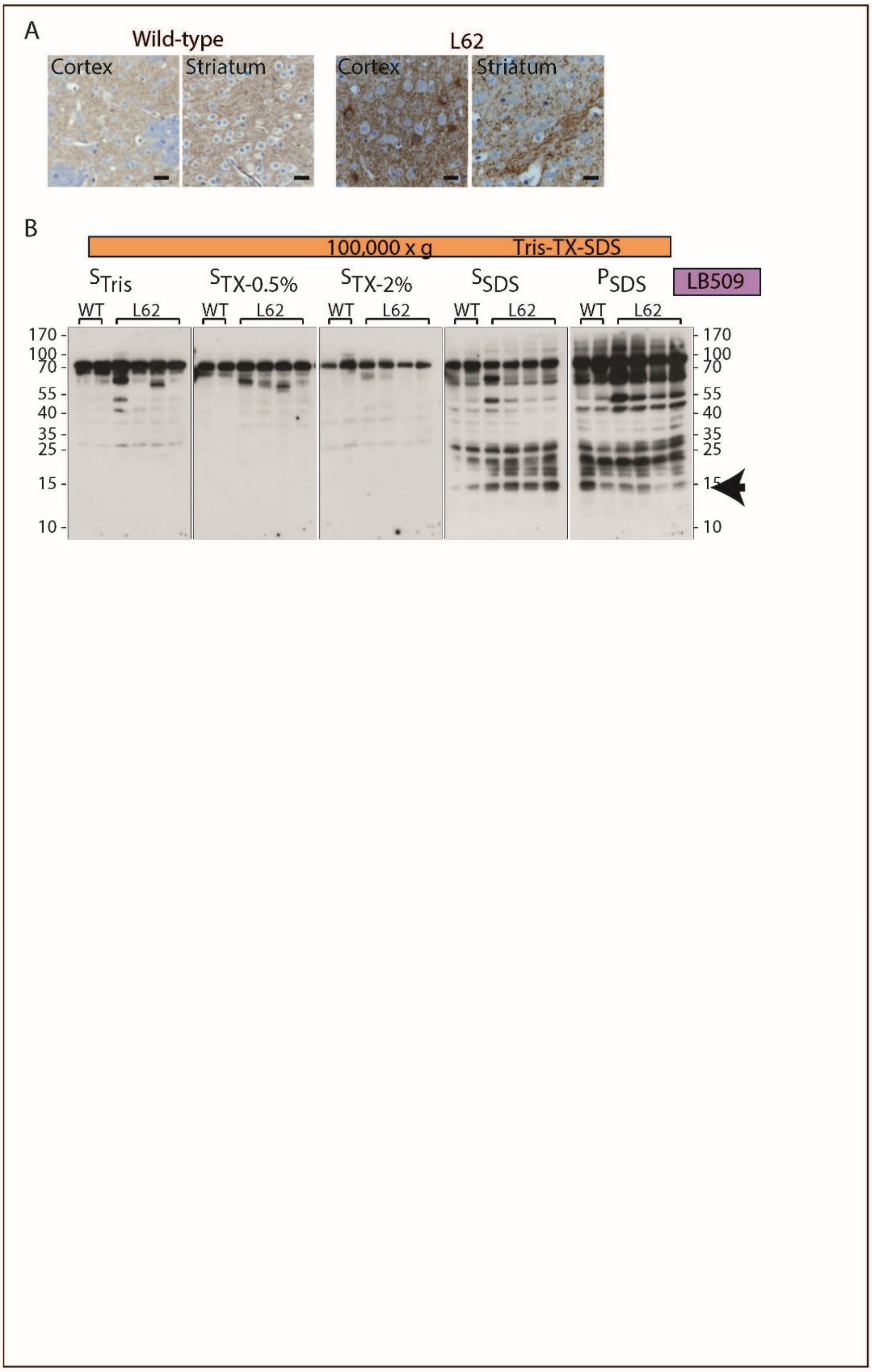

Supplementary Figure 2: Specificity of anti-α-Syn antibodies in L62 mice. Representative immunoblots for wild-type (WT) and L62 mice using (A) the mAb 211, (B) mAb 3H2897, (C) the polyclonal antibody C20, and (D) the mAb 4D6. Proteins were extracted from brain tissue using urea and separated by SDS-PAGE. Arrows indicate α-Syn monomer labelled at ~15kDa. # in (A) indicates recombinant α-Syn positive control.

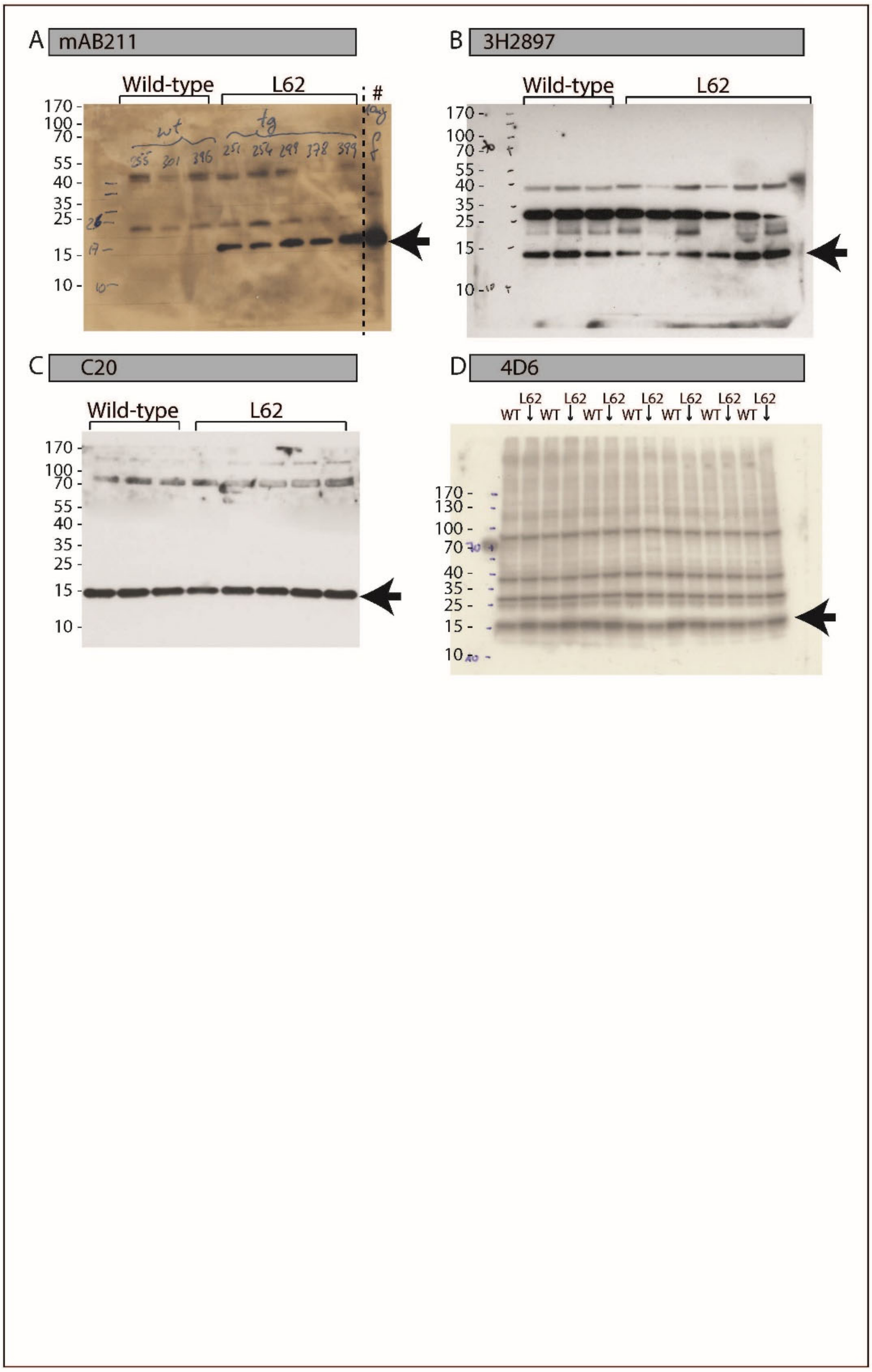

Supplementary Figure 3: Original uncropped images for immunoblots shown in Figure 1KLMNO.

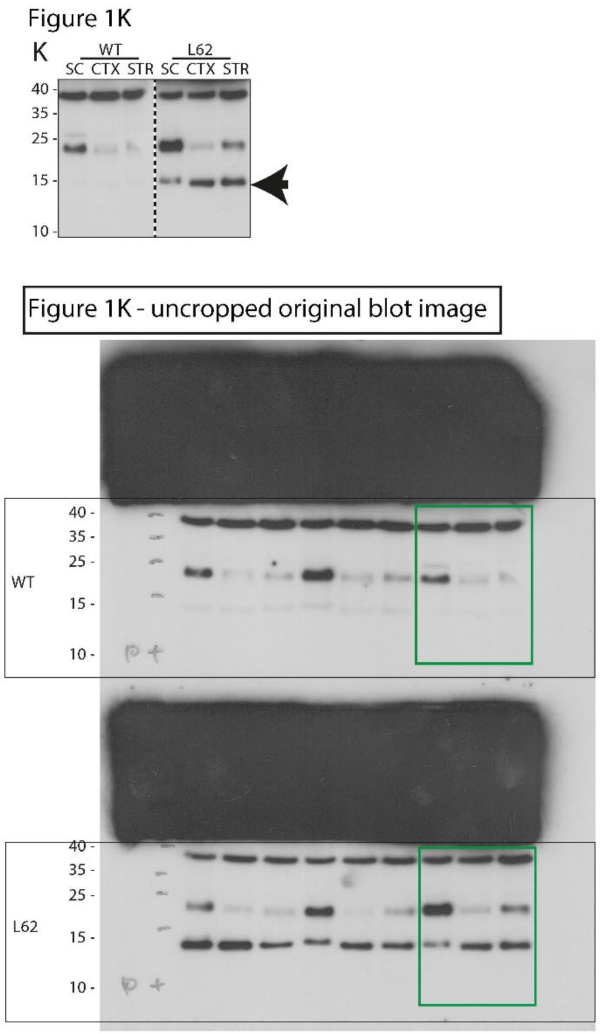

Supplementary Figure 3: continued.

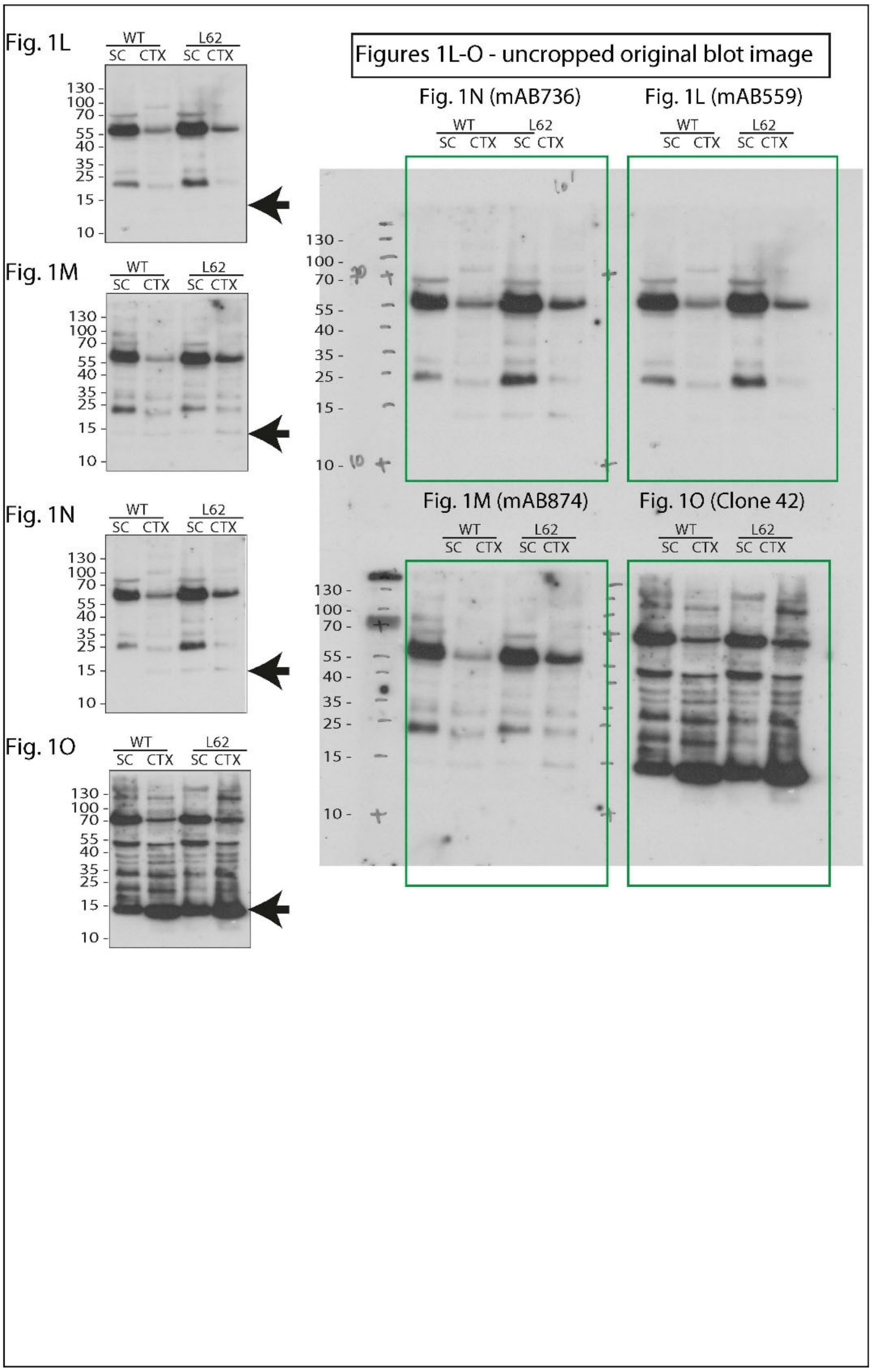

Supplementary Figure 4: Original uncropped images for immunoblots shown in Figures 3A, 3B and 3C.

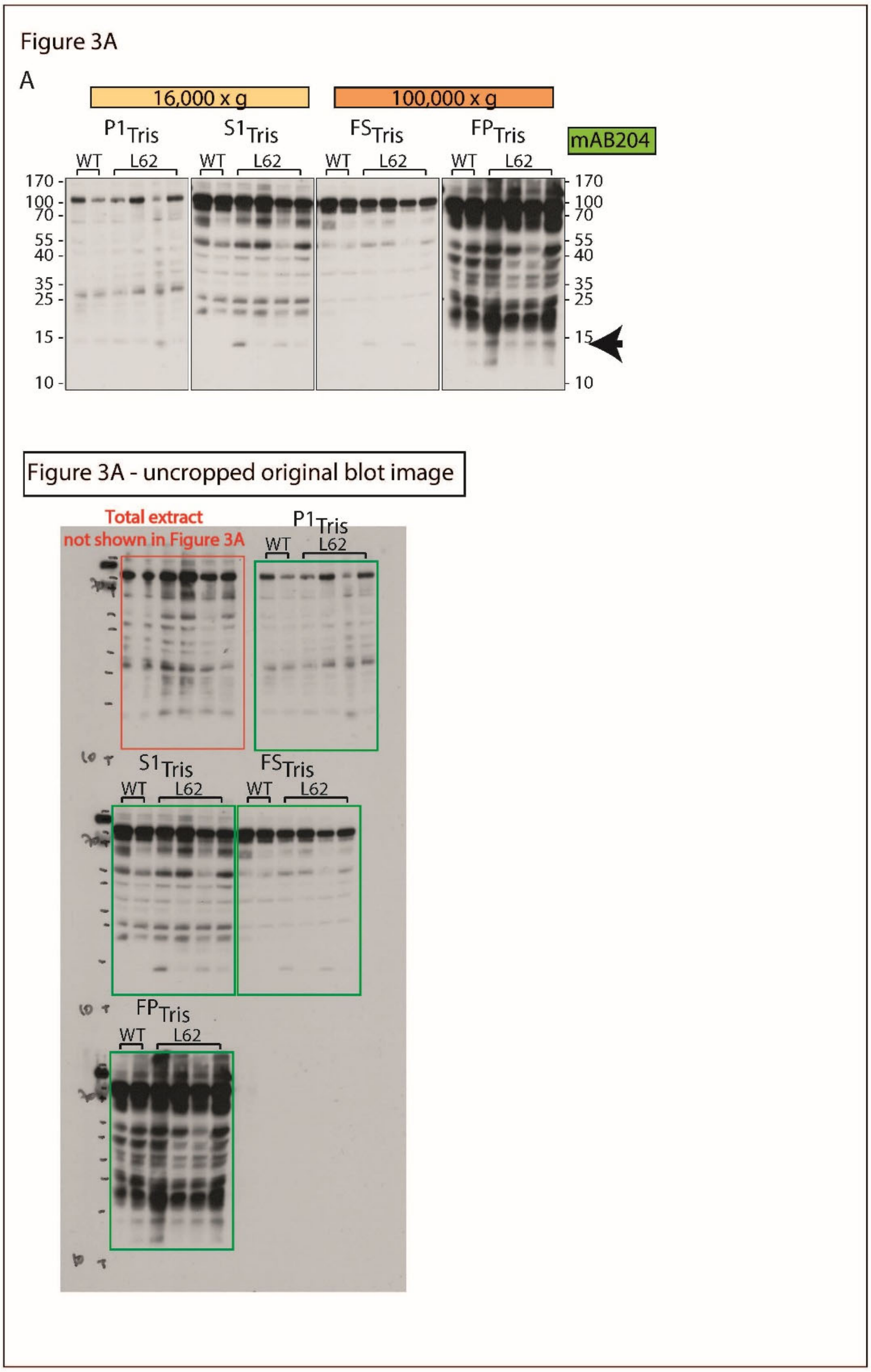

Supplementary Figure 4 – continued.

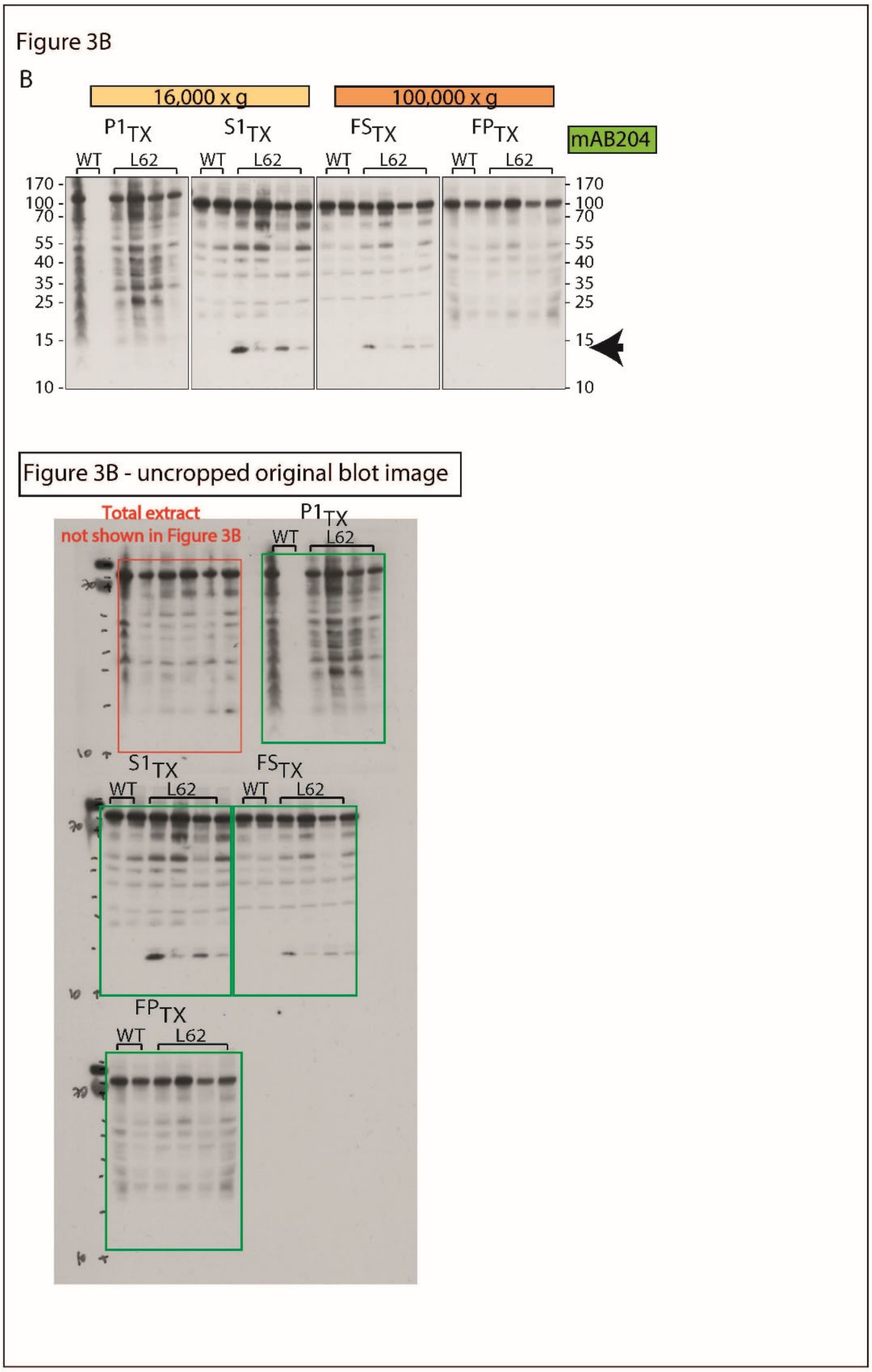

Supplementary Figure 4 – continued.

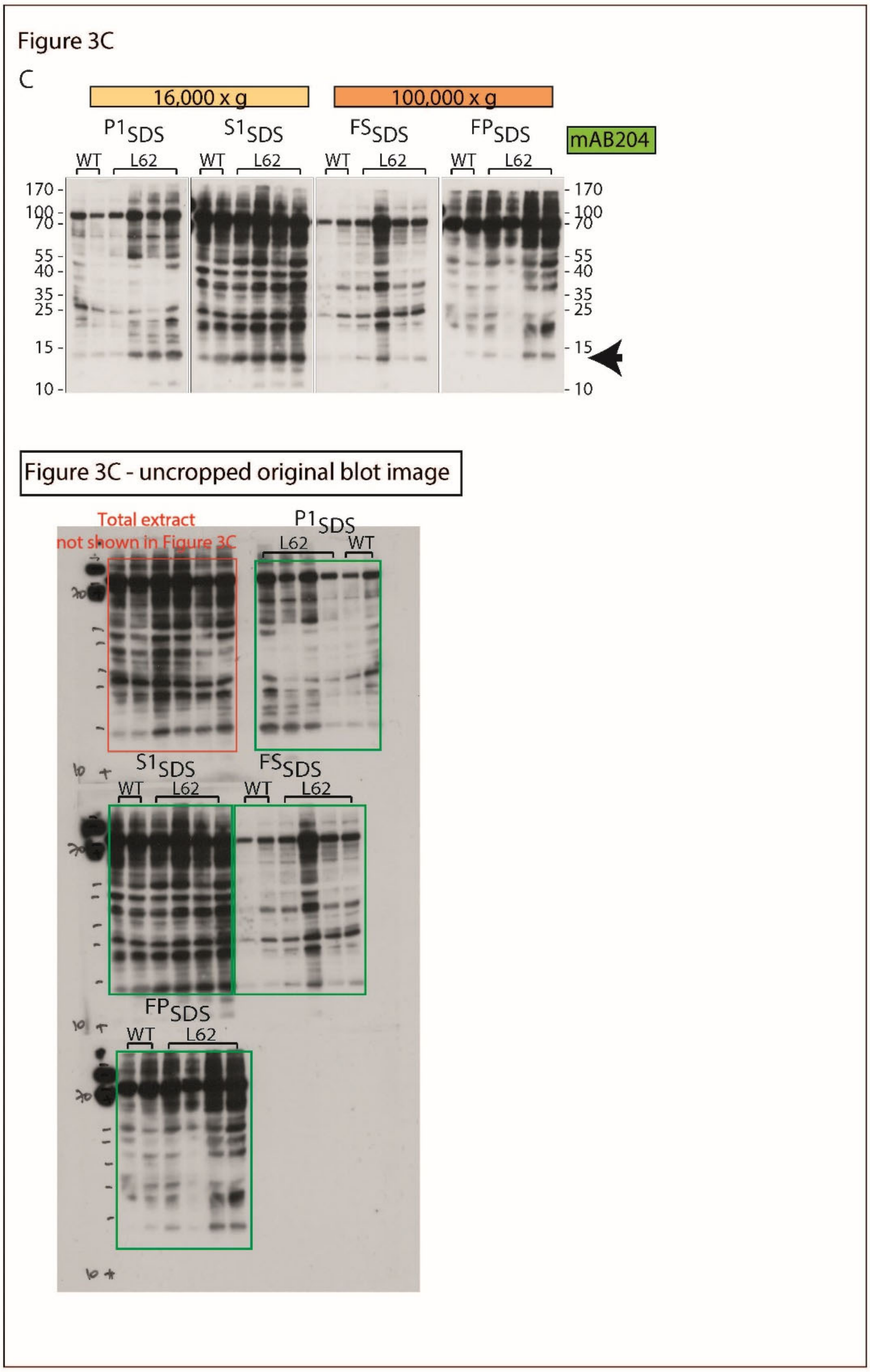

Supplementary Figure 5: Original uncropped images for immunoblots shown in Figures 4A and 4B.

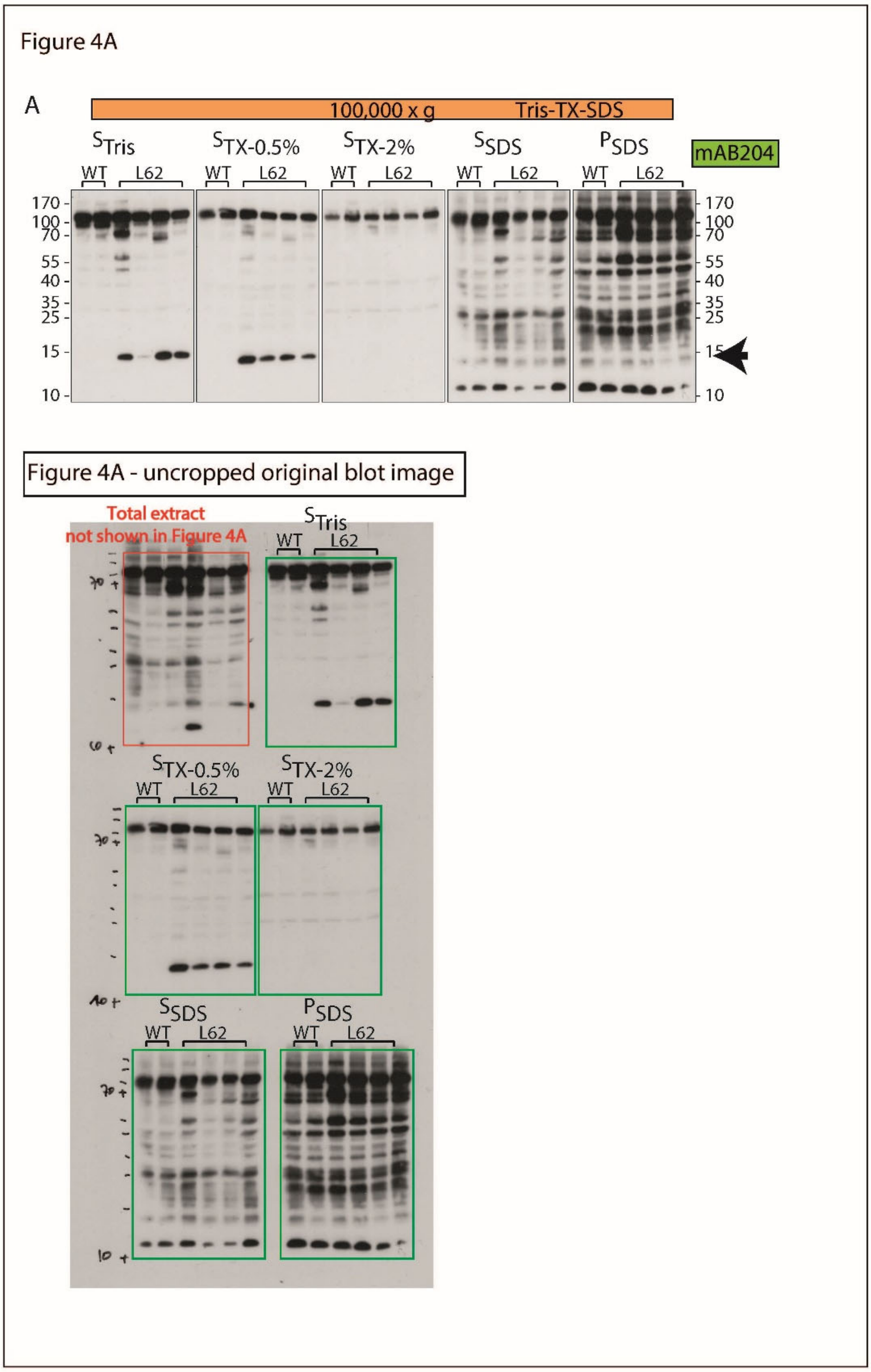

Supplementary Figure 5 – continued.

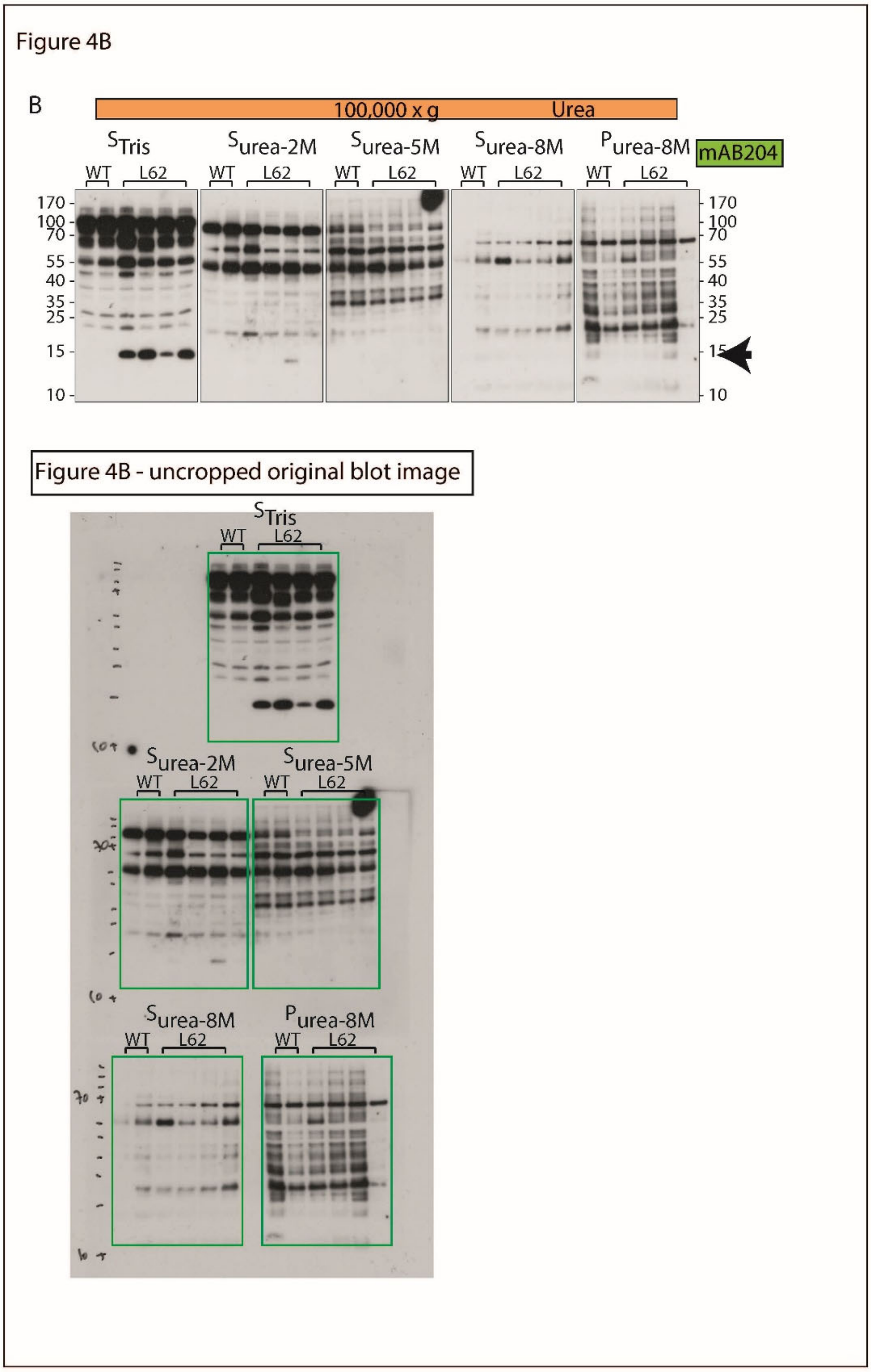

Supplementary Figure 6: Original uncropped images for immunoblots shown in Supplementary Figure 1B.

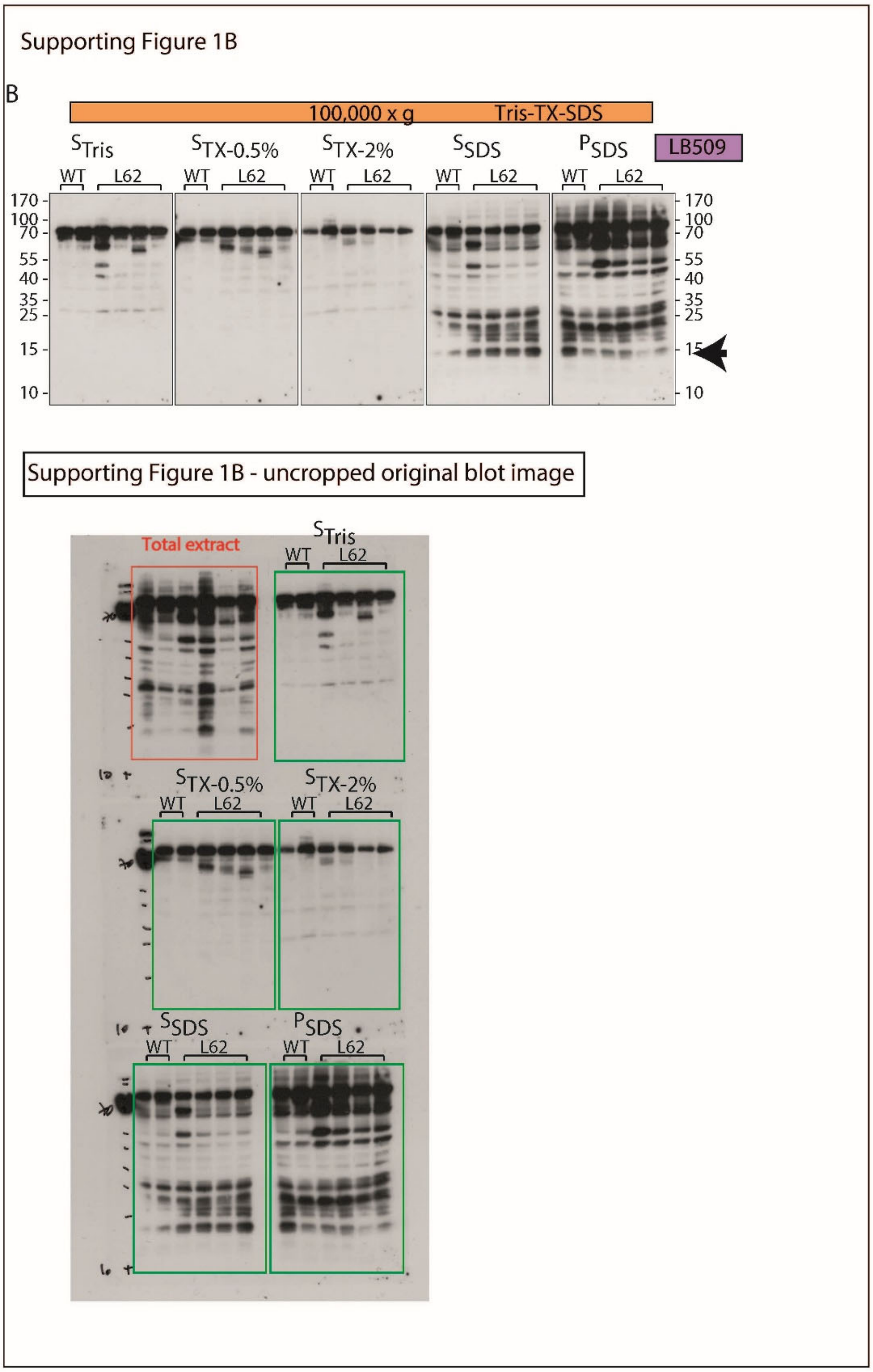

Supplement: Supplementary file 1 — Supplementary Figures. [file 41598_2024_56735_MOESM1_ESM.pdf]
